# Uncovering Latent Structure in Gliomas Using Multi-Omics Factor Analysis

**DOI:** 10.3390/genes17050540

**Published:** 2026-05-01

**Authors:** Catarina Gameiro Carvalho, Alexandra M. Carvalho, Susana Vinga

**Affiliations:** 1 Instituto Superior Técnico, Universidade de Lisboa, 1049-001 Lisbon, Portugal; catarinagameiroc@tecnico.ulisboa.pt; 2 Instituto de Telecomunicações, Instituto Superior Técnico, Universidade de Lisboa, 1049-001 Lisbon, Portugal; alexandra.carvalho@tecnico.ulisboa.pt; 3 Instituto de Engenharia de Sistemas e Computadores-Investigação e Desenvolvimento (INESC-ID), Instituto Superior Técnico, Universidade de Lisboa, 1000-029 Lisbon, Portugal; 4 IDMEC, Instituto Superior Técnico, Universidade de Lisboa, 1049-001 Lisbon, Portugal

**Keywords:** multi-omics integration, prognostic biomarkers, survival analysis, molecular subtyping, latent factor model

## Abstract

Background: Gliomas are the most common malignant brain tumors in adults, characterized by a poor prognosis. Although the current World Health Organization (WHO) classification provides clear guidelines for classifying oligodendroglioma, astrocytoma, and glioblastoma patients, significant heterogeneity persists within each class, limiting the effectiveness of current treatment strategies. With the increasing availability of large-scale multi-omics datasets resulting from advancements in sequencing technologies and online repositories that provide them, such as The Cancer Genome Atlas (TCGA), it is now possible to investigate these tumors at multiple molecular levels. Methods: In this work, we apply integrative multi-omics analysis to explore the interplay between genomic (mutations), epigenomic (DNA methylation), and transcriptomic (mRNA and miRNA) layers. Our approach relies on Multi-Omics Factor Analysis (MOFA), a Bayesian latent factor analysis model designed to capture sources of variation across different omics types. Results: Our results highlight distinct molecular profiles across the three glioma types and identify potential relationships between methylation and genetic expression. In particular, we uncover novel candidate biomarkers associated with survival as well as a transcriptional profile associated with neural system development. Conclusions: These findings may contribute to more personalized therapeutic strategies, potentially improving treatment effectiveness and survival outcomes in this disease.

## 1. Introduction

Advancements in sequencing technologies have significantly expanded our understanding of biological systems, leading to the generation of large amounts of high-dimensional data [1]. Various online platforms now provide access to omics data from different diseases, offering valuable resources for biomedical research [1]. Effectively analyzing all of this information requires computational methods capable of handling large datasets. Additionally, to further improve comprehension, the integration of multiple layers of biological data has become crucial, as it leverages interconnected information across different biological domains [2]. In this context, multi-omics integration analysis plays a crucial role, as it enables a comprehensive view of tumor biology by combining information from multiple molecular layers, including genomics, transcriptomics, epigenomics, and proteomics [1,2].

Many multi-omics integration methods, with different underlying approaches, have been proposed and validated [3,4,5,6,7]. An important group of strategies can be classified as joint Dimensionality Reduction (jDR) methods that decompose each omics dataset into the product of a factors matrix (shared by all omics) and a loadings matrix unique for each omics dataset [3]. It encompasses correlation and covariance-based approaches aimed at maximizing the covariance or correlation between datasets, such as Canonical Correlation Analysis (CCA) [8] and its variants, as well as Factor Analysis (FA), which assumes latent variables capture the shared variance across the data. Factor analysis can further include Probabilistic/Bayesian Models, such as Multi-Omics Factor Analysis (MOFA) [9], or iCluster [10]. Other approaches include Similarity (Kernel)-based (KB) methods, where a similarity matrix is constructed from kernels and analyzed subsequently, and Network-based Integration (NB) methods [11], which represent data as networks, where the nodes are entities, and the edges are similarities and interactions. This approach typically incorporates molecular interaction networks or defines custom networks based on omics data. Although NB methods generally perform better due to their reliance on a priori biological knowledge [6], which helps reduce false discoveries, these resources are often incomplete or unavailable. Deep Learning (DL) models have gained significant attention in recent years due to their ability to effectively handle high-dimensional data and capture complex non-linear relationships [5]. Although they have demonstrated superior accuracy compared to traditional methods in downstream tasks such as classification and regression [12], their lack of interpretability remains a significant limitation, despite the integration of explainability techniques into some models [13,14].

Each of these approaches demonstrates the ability to uncover novel biomarkers and identify molecular signatures, which can then be verified in follow-up experimental and clinical studies [10], potentially offering new perspectives for therapeutic intervention. This is particularly critical in cancer research, defined by inherent heterogeneity and the diverse treatment responses observed between patients.

Gliomas, in particular, represent a challenging class of tumors due to their high degree of molecular heterogeneity [15] and are associated with poor survival outcomes [16]. Many classifications have been proposed and are continually revised. The WHO updated the classification in 2021 [17], introducing a revised system that incorporates molecular features in addition to histological features, enabling more accurate and clinically relevant stratification of glioma subtypes, which is crucial for the development of effective targeted therapies [18].

Adult-type diffuse gliomas are classified into three subtypes: *astrocytoma* (grades II, III, IV), *oligodendroglioma* (grades II, III), and *glioblastoma* (grade IV) [19]. The term Lower-Grade Gliomas (LGGs) typically refers to the first two, highlighting their lower aggressiveness compared to glioblastoma (GBM). The mutations in the genes *IDH1* or *IDH2*, present in astrocytomas and oligodendrogliomas, are in fact associated with a better prognosis [19]. What distinguishes astrocytoma from oligodendroglioma, within LGGs, is the presence of the 1p/19q codeletion in oligodendroglioma patients. On the other hand, GBM is an *IDH*-wildtype tumor characterized by a gain of chromosome 7 and loss of chromosome 10 (+7/−10), along with other key molecular alterations, including *EGFR* amplification and/or *TERT* promoter mutations. The presence of morphological features such as necrosis, microvascular proliferation, and mitotic activity is also responsible for a higher grade classification [19].

Despite significant advancements in understanding gliomas at the molecular level, many aspects of their biology remain poorly understood, as evidenced by the poor survival rates associated with these tumors. A key contributing factor is the extensive molecular heterogeneity observed in these tumors [19]. The existing knowledge gap highlights the need for further investigation into the molecular mechanisms underlying glioma pathogenesis. A deeper understanding of these complexities is essential for developing more effective, personalized treatment strategies tailored to the unique molecular characteristics of individual patients [18].

## 2. Materials and Methods

### 2.1. Data Availability

The integration in the present study focused on several omics layers, specifically genomics (mutations), as they are key drivers of glioma heterogeneity, along with transcriptomics (mRNA and miRNA) and epigenomics (DNA methylation). The data were obtained from The Cancer Genome Atlas (TCGA) under the project names “TCGA-GBM” and “TCGA-LGG”.

The mutations dataset was downloaded using the RTCGAToolbox (version v2.32.1) [20] package, while the others were obtained using the TCGAbiolinks (version v2.30.4) [21] package. The downloaded data included binary mutation profiles, count-based transcriptomics, and beta values representing the methylation proportion of each probe. These last two datasets were provided in the *summarizedExperiment* format, which contains not only the expression matrix but also feature metadata, including gene annotations and additional biological information, such as associated chromosomal locations and corresponding gene names. Clinical data, including patient demographics (age, sex) and survival information, were also extracted. Its summary is in Table 1.

The ground-truth glioma labels used were from the study [22], where the TCGA labels were updated according to the most recent WHO guidelines from 2021. The labels assigned were: “Astrocytoma”, “Glioblastoma”, “Oligodendroglioma” or “Unclassified”. Table 2 highlights significant discrepancies between the labels assigned by the WHO and those provided by TCGA for the same patients.

All the code is available at https://github.com/sysbiomed/MOFA-in-Gliomas (accessed on 15 April 2026) to ensure reproducibility and modularity.

### 2.2. Data Preprocessing

To address missing values in the DNA methylation dataset, features with 90% or more missing entries were excluded. Outliers and low-abundance features were filtered out across datasets to ensure data quality. Each dataset was transformed into continuous values, except for the mutations dataset, which remained binary. Specifically, in the epigenomics data, beta values were converted to M-values, which correspond to the methylation signal, while transcriptomics data were normalized, variance-stabilized, and transformed into logarithmic counts per million (log-CPM).

Low-variance features were filtered prior to integration to reduce computational complexity, balance the contribution of each omics layer, and improve model performance. For DNA methylation, only the top 2% most variable probes were retained, as this layer is both the highest-dimensional and contains a large proportion of features with near-zero variance that contribute little to the model. For mRNA, the top 50% most variable features were kept, while for miRNA, 80% of features were retained to compensate for the smaller size of this omic.

Lastly, samples from the four omics datasets were intersected, yielding a final cohort of 318 patients. Among them, 142 are classified as astrocytoma, 80 as glioblastoma, and 84 as oligodendroglioma, with 12 samples left unclassified. Sex and age information were not available for only one patient. Regarding survival data, it is complete for 315 patients.

**Figure 1:**
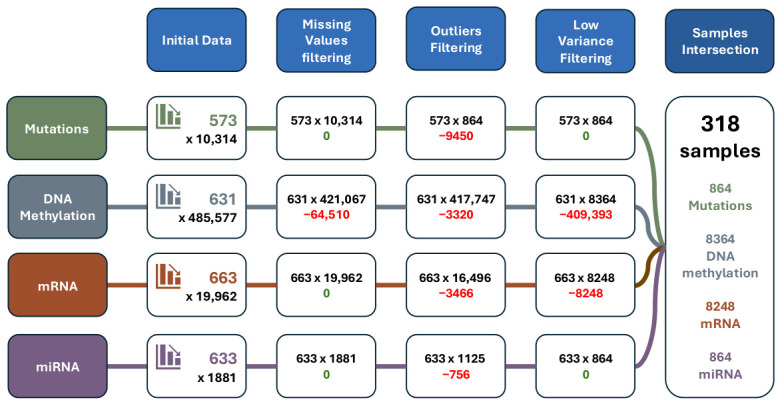
Schema illustrating the number of samples and features per omics layer at each preprocessing step, from the initial download of the TCGA dataset to the final filtered dataset used for MOFA.

A summary of the number of samples and features per omics layer at each preprocessing step is in Figure 1.

### 2.3. Multi-Omics Factor Analysis (MOFA)

MOFA is a factor analysis method that operates within a probabilistic Bayesian framework [9]. Given *M* omics datasets represented by $X^{m}$ with dimension $n\times p_{m}$, where m=1,2,…,M, *n* is the number of observations and $p_{m}$ is the number of features in the *m*-th omics dataset. The goal of MOFA is to factorize each matrix into a shared set of *K* latent factors:

(1) $$X^{m}=ZW^{mT}+\epsilon^{m},$$

where $Z_{n\times K}$ is called the factors matrix, shared across all of the omics, capturing the low-dimensional latent variables, while $W_{p_{m}\times K}^{m}$ is the loadings matrix that relates the high-dimensional space to the low-dimensional representation. The residual noise, $\epsilon^{m}$, captures variability not explained by the model and allows for heteroscedasticity across features, with each feature having variance $1/\tau_{d}^{m}$.

The prior for *Z* is a standard normal distribution, ensuring that the latent factors are normally distributed. For the loadings $W^{m}$, MOFA incorporates two types of sparsity constraints: one on the factors (View- and factor-wise sparsity) and another on the weights (Feature-wise sparsity). The latter is based on the assumption that biological sources of variability are typically sparse, meaning that only a small number of features are active. This results in the prior given by:

(2) $$p\left({\hat{w}}_{d,k}^{m},s_{d,k}^{m}\right)=N\left({\hat{w}}_{d,k}^{m}|0,\frac{1}{\alpha_{k}^{m}}\right)\;\mathrm{Ber}(s_{d,k}^{m}|\theta_{k}^{m}).$$

The term ${\hat{w}}_{d,k}^{m}$ represents the continuous weight component and $s_{d,k}^{m}\in \left\{0,1\right\}$ indicates if the weight is active.

Inference is performed using variational inference, where the posterior distribution is approximated through optimization by maximizing the Evidence Lower Bound (ELBO):

(3) $$\max_{q}ELBO=\max_{q}E_{q}[\log q\left(Z\right)+\log p\left(Z,X\right)].$$

In variational inference, the distribution q(Z) serves as an approximation of the true posterior p(Z|X,θ). The mean-field approximation further assumes that the variational distribution q(Z) factorizes over disjoint groups of variables, simplifying the optimization process. In MOFA, this translates into:

(4) $$q\left(Z,S,\hat{W},\alpha ,\tau ,\theta \right)=q\left(Z\right)\;q\left(\alpha \right)\;q\left(\theta \right)\;q\left(\tau \right)\;q(S,\hat{W}).$$

MOFA offers great flexibility by supporting different priors for the residual noise, Gaussian, Bernoulli, or Poisson, thereby handling continuous, binary, and count data. However, binary and count data are often not well modeled by these distributions. Count data is usually very sparse, making it preferable to apply data transformations to approximate a Gaussian distribution. Additionally, MOFA naturally accommodates missing values by excluding them from the likelihood computation, thus not affecting the update equations.

MOFA was applied in this work using the R package v2.15 MOFA2. To ensure robustness in MOFA model selection, 10 independent runs were performed, each initialized with a different random seed. The model was configured with sparsity constraints enabled, and the number of factors was not predefined; instead, factors explaining less than 5% of the variance were automatically discarded. The likelihoods were assigned based on data characteristics, with a Gaussian distribution applied to most omics data, while binary likelihoods were used for genomic features. The convergence mode was set to “medium”, stopping when the change in delta ELBO reached 0.00005%. After training, the best model was selected using ELBO-based optimization.

### 2.4. Differential Gene Expression Analysis (DGE)

DGE analysis was performed to identify the most significant genes distinguishing glioma subtypes in mRNA and DNA methylation data. For mRNA, theedgeR package (version v4.0.16) [23] was used with a threshold of 0.05 for the False Discovery Rate (FDR) and regarding Fold-Change (FC), $|\log_{2}\mathrm{FC}|>1$. For methylation, CHAMP was applied with FDR < 0.05 and a change in mean beta values (Δβ) greater than 0.3.

### 2.5. Gene Set Enrichment Analysis (GSEA)

GSEA was performed on ranked gene features from MOFA, using the ReactomePA (version v1.46.0) [24] package for mRNA, returning significantly enriched Reactome pathways. Each significant pathway was then mapped to its corresponding top-level (root) category by following the official Reactome hierarchy. Top-level pathway annotations were obtained from a reference file available on the Reactome website. When not available in this reference file, the top-level pathways were retrieved using an R interface to the Reactome API [25]. This mapping was used to summarise the distribution of significant pathways across broad biological categories, enhancing the interpretability of the factor-specific enrichment results. This summary is descriptive and does not test enrichment of the top-level categories themselves.

The missMethyl package (version v1.36.0) [26] was used for DNA methylation analysis to correct for bias caused by varying CpG coverage across genes. The enrichment analysis focused on Gene Ontology (GO) pathways, specifically Biological Processes (BP). Only pathways containing between 5 and 500 genes were considered for testing, using a significance threshold of p<0.05, with multiple testing correction applied via the Benjamini–Hochberg (BH) procedure. To focus the analysis on regions with potential regulatory impact, we further restricted the input to CpG sites in promoter-associated regions, specifically those annotated as *TSS1500*, *TSS200*, or *1stExon*.

### 2.6. Survival Analysis

To assess statistical differences between two Kaplan–Meier survival curves, the log-rank test was performed with groups defined by median gene expression. This analysis was conducted using the survival package (version v3.8.3) [27].

## 3. Results

### 3.1. Overview of the Model

Applying MOFA resulted in the identification of four factors. The first factor accounts for 80% of the total variance, capturing co-variation across all datasets, suggesting its strong relevance. Factor 2 primarily explains variance within the mRNA assay, while factor 3 captures variation across all datasets except for miRNA.

**Figure 2:**
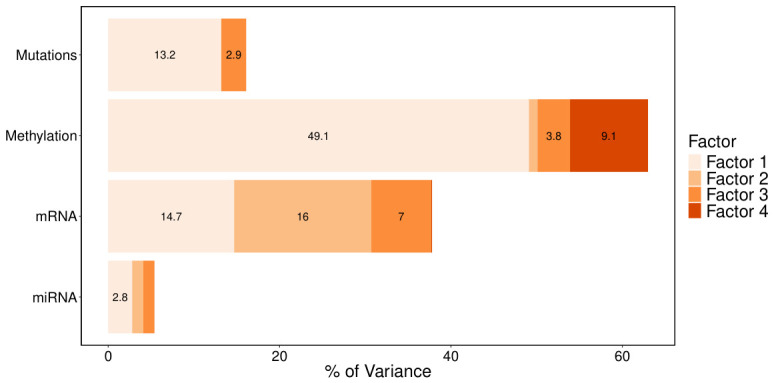
Variance decomposition by omics data type and latent factors. Variance decomposition by omics data type and latent factors. Only variance values for factors explaining more than 2% of the variance within an omics dataset are shown.

Notably, the miRNA view explains only 5.17% of the variance captured by the model (Figure 2). To assess whether miRNA inclusion influences the inferred latent structure, we refit MOFA excluding the miRNA view; the variance decomposition and factor structure across the remaining omics layers were largely unchanged. However, miRNA was retained in the model due to its well-established regulatory role in post-transcriptional gene expression, which is directly relevant to the biological question under investigation.

Consequently, factors 1 and 3 appear to capture relevant general information of gliomas, while factor 2 reflects a specific gene expression pattern.

Factor 4 captured sex-associated variation and was driven primarily by the DNA methylation view. CpG sites with the largest absolute loadings mapped predominantly to the X chromosome, and Factor 4 values clearly separated samples by sex, consistent with X-inactivation-related methylation patterns. Because this factor reflects demographic variation rather than glioma-specific heterogeneity, we excluded it from downstream clustering and biological interpretation. We note that excluding sex-chromosome CpGs prior to model fitting is a reasonable alternative preprocessing strategy in DNA methylation analyses.

**Figure 3:**
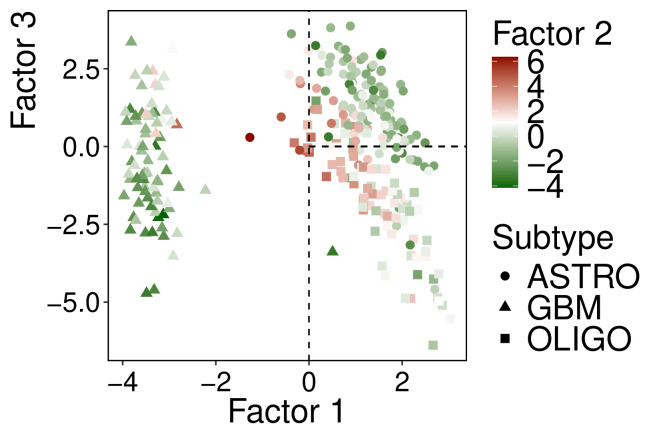
Latent space projections of classified samples across MOFA factors 1 and 3, shaped by subtype and colored by factor 2 values. Dashed lines indicate the vertical line at Factor 1 = 0 and the horizontal line at Factor 3 = 0 (extending from Factor 1 = 0 onwards).

Sample projections of the selected factors are shown in Figure 3.

**Figure 4:**
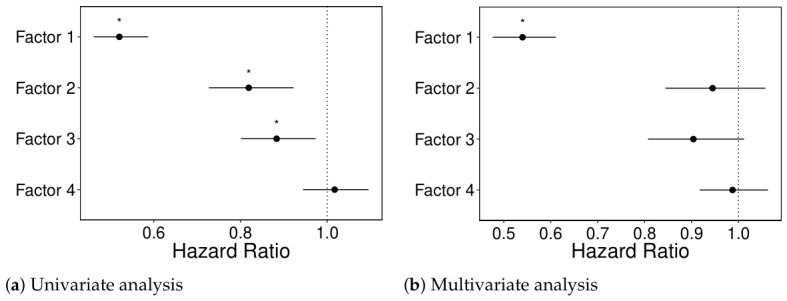
Hazard ratios and corresponding 95% confidence intervals for the four MOFA-derived factors. Statistically significant associations (p<0.05) are marked with an asterisk (*). (**a**) Univariate analysis for each factor separately. (**b**) Multivariate analysis including all factors simultaneously.

Fitting a univariate Cox proportional hazards model to each factor individually revealed that the first three factors were significantly associated with survival at the 0.05 significance level. However, when all factors were included simultaneously in a multivariate model, only the first factor remained statistically significant. Univariately, the first factor has a hazard ratio of approximately 0.52, indicating that a one-unit increase is associated with a 48% reduction in the hazard of death. This result is consistent with the known aggressiveness of GBM, suggesting that this factor may capture relevant biological signals related to poor prognosis. The results are presented in Figure 4.

#### 3.1.1. Features Selected

**Figure 5:**
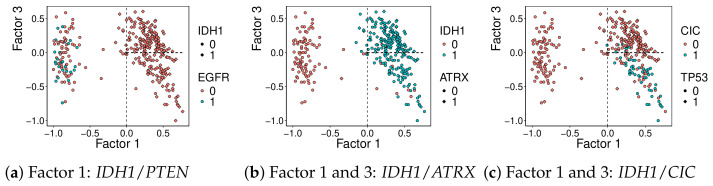
Projection of samples on factors 1 and 3, colored and shaped by driver mutation status. (**a**) Mutations associated with factor 1 (*IDH1* positive, *PTEN* negative). (**b**) Mutations positively associated with both factor 1 (*IDH1*) and factor 3 (*ATRX*). (**c**) Mutations associated with *IDH1* and *CIC* with factors 1 and 3, respectively.

The loadings matrix for mutations and miRNA is notably sparse. It is observed that, for the mutation loadings matrix, factor 1 is strongly associated with gene *IDH1* on the positive side, while it selects *PTEN* and *EGFR* negatively. Factor 3 highlights *ATRX* and *TP53*, while it shows a negative association with *CIC*. Figure 5 shows the clear separation of mutation profiles by these two factors.

Considering miRNA, for factor 1 (only expressed in this factor), the following genes *ENSG00000278783*, *ENSG00000207575*, *ENSG00000266174* were selected, corresponding to the genes *MIR6071*, *MIR649* and *MIR4666A*, respectively, associated with a positive loading.

The loading matrices for mRNA and DNA methylation were not particularly sparse. Therefore, for each of the three factors (1, 2, and 3), the top 30 features with the highest absolute loadings were selected to facilitate a more focused and interpretable analysis. The absolute value of the loadings was considered because, unlike mutations, both high and low values in these omics layers (mRNA and DNA methylation) can carry meaningful biological information. Despite this, the selected features generally exhibited consistent sign patterns across factors. All methylation features selected for factor 1 had positive loadings, whereas those for factor 3 had negative loadings. Interestingly, eight probes selected by factor 3 are assigned to the gene *ISM1*. For mRNA, the selected features in factor 1 were mostly negative (except for three genes, *RICTOR*, *MARCHF8*, and *BMP2*), while those in factors 2 and 3 were all positive. None of the genes associated with the CpGs (given by *Illumina 450k* platform) selected are in the set of mRNA features selected.

#### 3.1.2. GSEA Results

**Figure 6:**
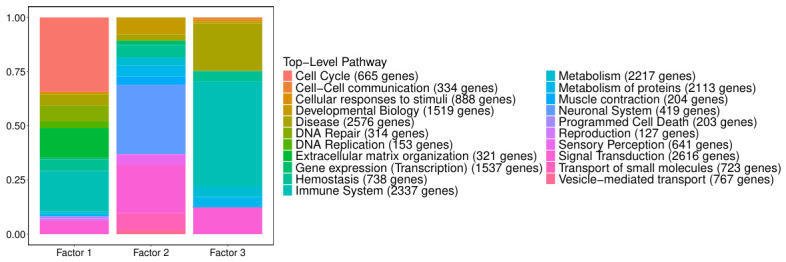
Distribution of significantly enriched Reactome pathways across top-level (root) categories, based on ReactomePA GSEA of ranked mRNA feature loadings across MOFA factors. Pathway labels indicate the total number of unique genes annotated to each top-level category in the Reactome hierarchy (shown in parentheses).

Given the overall directionality of the loadings of the features selected, GSEA was performed accordingly. The results of the top-level Reactome pathways for the mRNA features selected are in Figure 6.

Overall, factor 1 captures a heterogeneous mix of biological processes, with enrichment in the immune system, cell cycle, and other functional categories such as extracellular matrix organization, whereas factor 2 reveals a distinct functional profile, pointing to a key role of the neuronal system. Also, signal transduction processes appear to play an important role in this factor. Factor 3 is specifically associated with immune-related functions. It also has a large proportion of pathways attributed to the ‘Disease’ top-level category, most of which correspond to cancer.

**Figure 7:**
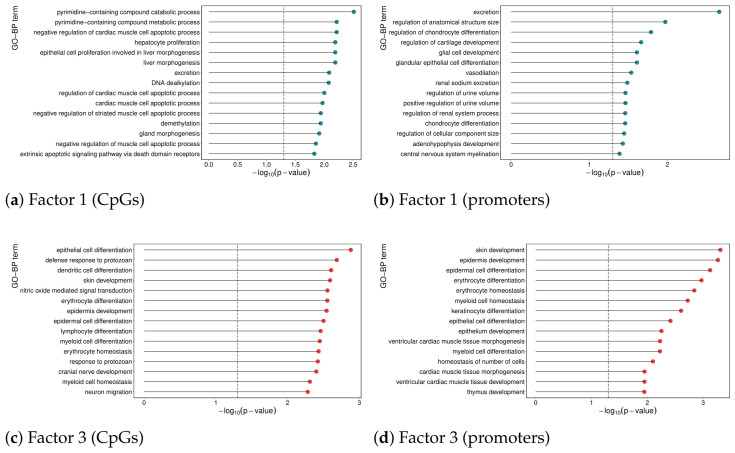
Gene ontology biological process (GO-BP) enrichment results for DNA methylation features associated with factors 1 and 3. (**a**,**b**) Positive associations with factor 1; (**c**,**d**) negative associations with factor 3. Only pathways with more than 5 and fewer than 500 genes were tested. Significance threshold: p<0.05.

The top 15 enriched Gene Ontology biological processes associated with factors 1 and 3, based on the overall sign of their most important features, are shown in Figure 7.

The CpGs associated with factor 1 are predominantly enriched in pathways related to cell apoptosis, with additional involvement in metabolic and epigenetic processes. Focusing on promoter regions, methylation changes at factor 1 CpGs are primarily associated with pathways involved in tissue development and cell differentiation, which is especially relevant given the glial origin of LGG (the ones with factor 1 positive). Additionally, several homeostasis-related pathways are present.

The pathways enriched by factor 3 highlight a strong theme around cell differentiation and immune system function. These include the development of various cell types, such as epithelial, epidermal, dendritic, erythrocyte, lymphocyte, and myeloid cells. A similar pattern is observed in promoter regions, and given that the same biological functions are enriched among factor 3 genes, this suggests that methylation at these CpG sites is likely associated with gene silencing.

### 3.2. Interpretation and Evaluation of the Findings

The CpGs along with the genes *RICTOR*, *MARCHF8*, and *BMP2*, selected by factor 1, were found to be upregulated in LGG samples. In contrast, the remaining 27 genes exhibited higher expression levels in GBM samples. Notably, increased CpG methylation and mRNA gene expression were significantly associated with improved survival.

The selected factor 3 genes are consistently upregulated in astrocytoma compared to oligodendroglioma samples, supporting the hypothesis that factor 3 is involved in the separation of the LGGs. An exception is observed for three genes—*BHLHE41*, *ADRB2*, and *ATP8B4*—which, although statistically significant, display log_2_ fold-change values slightly below the threshold of 1. Within the LGG group, all but five are significantly associated with survival. GBM samples do not show a consistent pattern in the features associated with factor 3, and, in fact, no gene shows significant differential expression between astrocytomas and GBM.

The genes associated with factor 2 seem to be linked to a less aggressive tumor phenotype. In fact, they are consistently downregulated when comparing astrocytomas and oligodendrogliomas to glioblastomas, suggesting a potential role in LGGs. However, no significant expression difference is observed between astrocytomas and oligodendrogliomas, likely because these genes are shared across specific subgroups within both tumor types. Indeed, a high expression of these genes within LGG is associated with better survival significance, except for three genes: *CAMKK1*, *DMTN*, and *STX1A*.

**Figure 8:**
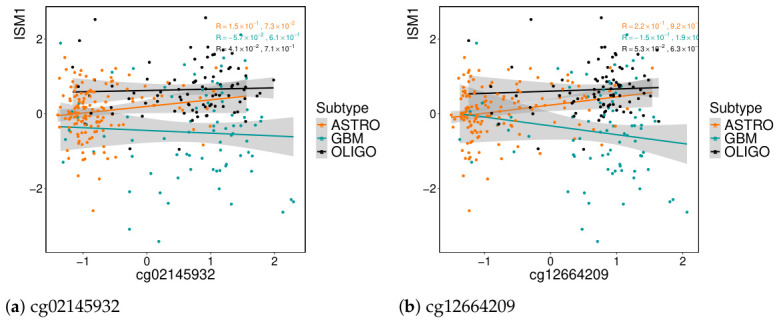
Correlation between methylation and gene expression for two probes annotated to *ISM1*. The data corresponds to the input matrices used in MOFA. Probe annotations are based on the Illumina 450K array. (**a**) cg02145932; (**b**) cg12664209.

The genes and probes associated with the same factor appear to be connected, as evidenced by strong Pearson correlations among them and consistently low correlations with features from other factors. However, regarding gene regulation, the association of each probe with its gene is not that direct. From factor 3, eight probes were assigned to *ISM1* but do not seem to be regulating the gene, as evidenced in Figure 8 for two of those probes.

**Figure 9:**
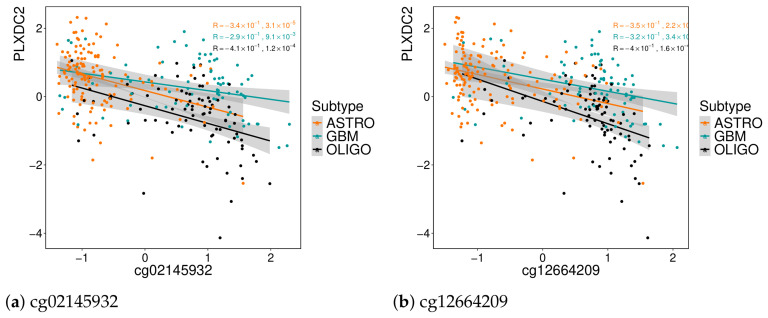
Correlation between methylation and gene expression for two probes annotated to *PLXDC2*. The gene *PLXDC2* was also selected by this factor. Correlations are computed using the input matrices for MOFA. Probe annotations are based on the Illumina 450K array. (**a**) cg02145932; (**b**) cg12664209.

Understanding how DNA methylation affects gene expression is highly complex [28,29]. The effect of methylation is generally stronger when an entire region is methylated. Since eight probes that were selected are associated with the same gene (*ISM1*), they may have a significant impact. By analyzing their correlation with the genes selected by factor 3, they show almost double the correlation, showing slightly stronger associations with the genes *SLC2A5*, *BLNK*, *TMEM119*, and *PLXDC2*. For the last gene mentioned, the correlation plots are in Figure 9, with the same two probes given as an example above.

### 3.3. Uncover MOFA-Derived Profiles

The MOFA factors appeared informative: factor 1 distinguished the GBM and LGG groups, whereas factor 3 revealed heterogeneity within the LGG group. Factor 2 was associated with high expression of neural system-related genes, suggesting an additional molecular profile beyond the three tumor labels. It was hypothesized that this profile might be associated with lower aggressiveness in the LGG group, prompting further investigation.

**Figure 10:**
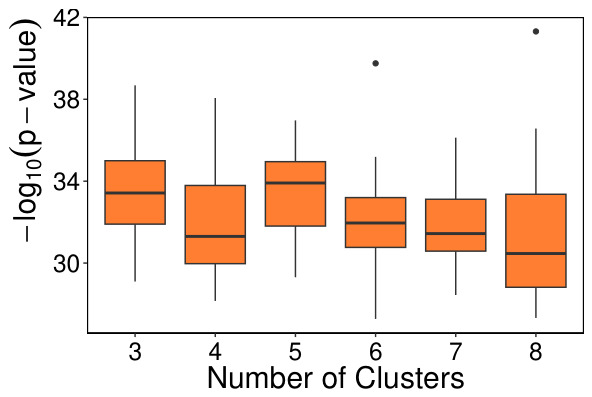
Distribution of the $-\log_{10}$ (*p*-values) across different numbers of clusters tested.

To explore this hypothesis, k-means clustering was applied using the three factors as input. The goal was to identify the number of clusters that best correlated with patient survival, specifically based on the *p*-values from the log-rank test. To account for variability due to k-means initialization, the analysis was repeated 30 times using 80% of the samples in each iteration, ensuring a balanced representation of censored and uncensored patients. The distribution of these *p*-values is shown in Figure 10.

**Figure 11:**
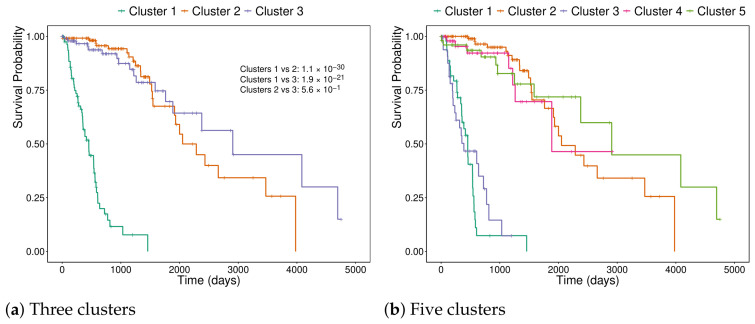
Kaplan–Meier survival curves for 318 patients stratified by the three MOFA factors using k-means clustering. (**a**) Three clusters and pairwise log-rank *p*-values. (**b**) Five clusters.

**Figure 12:**
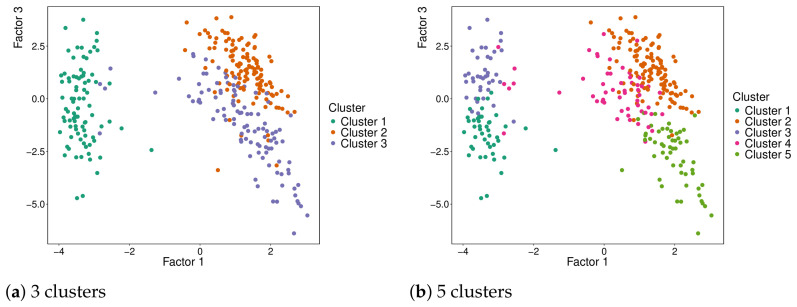
Sample projections of 318 patients according to cluster assignments. (**a**) *k* = 3 clusters. (**b**) k=5 clusters.

The optimal number of clusters identified was five, although the difference in performance compared to using 3 clusters (the baseline) was not substantial. The survival curves for three and five clusters are in Figure 11, and the respective projections in MOFA factors 1 and 3 are in Figure 12. The pairwise *p*-values of the five cluster solutions are in Table 3.

Table 4 summarises sample distribution across the five clusters obtained with the described method, with proposed names GBM-1, ASTRO-2, GBM-3, MIX-LGG-4, and OLIGO-5. GBM cases belong mainly to clusters 1 and 3. Cluster 1 (GBM-1) contains the oldest patients and shows the highest percentage of deaths, possibly explaining its more aggressive survival profile compared to GBM patients in cluster 3 (GBM-3). This might be associated with a higher incidence of mutations in genes such as *EGFR*. Additionally, histological data reveal that patients in cluster 1 are mostly diagnosed with primary GBM, whereas cluster 3 includes fewer primary GBM and more astrocytomas, further supporting the difference in clinical outcomes between these two GBM-enriched clusters. The majority of astrocytoma cases are grouped in cluster 2 (ASTRO-2), whereas oligodendroglioma cases are mainly found in cluster 5 (OLIGO-5). Cluster 4 (MIX-LGG-4) shows a balanced mix of LGG subtypes, suggesting an intermediate profile. Compared with the 3-cluster solution, the 5-cluster setting yields clearer separation of LGG subtypes. In the 3-cluster case, one cluster grouped together 76 oligodendrogliomas and 25 astrocytomas, indicating limited resolution between these two subtypes. Notably, astrocytomas are often misclassified as GBM due to molecular similarities; however, this does not appear to occur with MOFA. In the 3-cluster solution, GBM samples were largely isolated into a single cluster, with only two samples assigned to the other two clusters.

DGE analysis was repeated using the mRNA and CpGs features selected by each MOFA factor and assessed within each cluster. The labels were changed to the respective group with the number of clusters at the end.

Factor 1 continues to capture features that separate high-grade gliomas from LGGs. Within GBM, factor 2 genes highlight strong differences between GBM-1 and GBM-3, with 24 Differential Expressed Genes (DEGs) between these two GBM groups. GBM-3 shares high expression of these genes with OLIGO-5 (0 DEG between them) and ASTRO-2 (1 DEG), suggesting that GBM-3 may exhibit a lower grade expression profile. Given the enrichment of these genes, GBM-3 could reflect a neural-like subtype of GBM, consistent with prior studies characterizing GBM subtypes [30]. The gene *SLC12A5*, which exhibits the highest loading in this factor, has previously been associated with the neural subtype of GBM, reinforcing this interpretation [30]. Moreover, the MIX-LGG-4 cluster exhibits a distinctive and unique expression signature, with 30 DEGs compared with all other clusters, which potentially correspond to a transitional or mixed phenotype that does not align clearly with classic LGG or GBM categories. Factor 3 primarily differentiates the LGG subtypes, especially OLIGO-5 from ASTRO-2. OLIGO-5 exhibits a large number of DEGs (up to 30) and differentially methylated CpGs (up to 24) when compared to ASTRO-2 and GBM subtypes, indicating a well-defined epigenetic and transcriptional identity. Interestingly, GBM-3 shows greater molecular similarity to ASTRO-2 and greater dissimilarity to OLIGO-5 in this factor. On the other hand, GBM-1 appears to share some methylation and gene expression characteristics with MIX-LGG-4, suggesting potential epigenetic convergence between these groups.

## 4. Discussion

GBM tumors represent the most aggressive glioma subtype, exhibiting higher cell proliferation and invasiveness compared to LGG [19]. In this study, we identified 27 genes overexpressed in GBM over LGG, predominantly linked to immune system activity, cell cycle regulation, and extracellular matrix organization—*FBXO17*, *RARRES2*, *JHY*, *PINLYP*, *AQP5*, *EVC2*, *XKR8*, *TOM1L1*, *EFEMP2*, *RBP1*, *CCDC8*, *FABP5*, *C9orf64*, *SH2D4A*, *CUL7*, *ARL9*, *CEP112*, *EID3*, *RAB34*, *RAB36*, *EMP3*, *SHROOM3*, *TSTD1*, *HDHD3*, *CMYA5*, *VASN* and *PRICKLE3*. Overexpression of *RAB34* is linked to poor prognosis, tumor invasion, and immune infiltration and is suggested as a possible immunotherapy target for glioma [31]. Though less studied, *RAB36* may share similar roles in vesicle trafficking, suggesting its potential relevance in GBM progression since they belong to the same RAB family of proteins.

The genes *RICTOR*, *MARCHF8*, and *BMP2* were found to be highly expressed in LGG tumors. All of them have been reported in the literature as overexpressed in LGG compared to GBM. The literature highlights the oncogenic role of the mTORC2 signaling pathway and its component *RICTOR* in GBM, with evidence that co-targeting *EGFR* and *RICTOR* leads to strong anti-tumor effects [32]. While *EGFR* alterations are known to activate the mTORC2 pathway, where *RICTOR* is a key component, this suggests that *RICTOR* overexpression in LGG may occur independently of *EGFR* mutation status, potentially reflecting alternative mechanisms of pathway regulation in LGG. In fact, this gene is in the Reactome pathway “Regulation of TP53 Expression and Degradation”, and its overexpression may contribute to enhanced degradation or reduced expression of *TP53*, a characteristic of the astrocytoma group. This set of 30 genes was identified as not only differentially expressed between the groups but also significantly associated with patient survival.

Three miRNA features were also found to be overexpressed in LGG: *MIR6071*, *MIR649*, and *MIR4666A*. Although they do not code for proteins, miRNAs play important regulatory roles in tumor biology, and their dysregulation impacts tumor suppressors and oncogenes [18]. *MIR6071* has been reported to be downregulated in GBM and is associated with tumor-suppressive functions, including the inhibition of cell proliferation, migration, and invasion. Notably, higher expression levels of *MIR6071* have been associated with a better prognosis in glioma patients [33]. The roles of *MIR649* and *MIR4666A* are less well characterized; however, both are predominantly expressed in brain tissues, suggesting a potential relevance in glioma biology.

Additionally, DNA methylation patterns in LGG appeared particularly relevant to the regulation of cell apoptosis, especially at promoter regions enriched for genes involved in homeostatic processes and glial cell development. Although the precise regulatory impact of these methylation events is unknown, a strong correlation was observed between the methylation levels of the selected probes—*cg24041541*, *cg19070139*, *cg13727691*, *cg20826224*, *cg12630147*, *cg05163329*, *cg25604326*, *cg16590910*, *cg01764954*, *cg03974423*, *cg00428601*, *cg21174055*, *cg12604950*, *cg18276016*, *cg03609308*, *cg19113375*, *cg01818121*, *cg22230604*, *cg20525712*, *cg16483867*, *cg01338255*, *cg01843034*, *cg19998675*, *cg22451910*, *cg01118078*, *cg12907983*, *cg21940568*, *cg26448489*, *cg08409074* and *cg25251738*—and the expression of the 30 genes mentioned above: methylation was negatively correlated with genes overexpressed in GBM and positively correlated with the three LGG-specific genes. Higher methylation levels of these probes are associated with improved patient survival.

A molecular sub-profile within GBM was identified, characterized by high expression of neural-related genes, and showed similarities to astrocytoma with respect to genetic expression and histological type. This less aggressive subgroup, found in younger patients, shows lower *EGFR* mutation rates. In fact, *EGFR* amplifications have been reported to define a much more aggressive tumor subpopulation, as it drives high-grade morphological features such as cell proliferation, invasion, and angiogenesis [15,18]. This is because, as a potent oncogene, *EGFR* amplification activates intracellular signaling pathways, including *PI3K/AKT/mTOR*, which are important regulators of cell growth and survival. Notably, *SLC12A5*, enriched in the cerebral cortex and linked to the neural GBM subtype [30], may play a vital role in the Central Nervous System. Other neuronal genes such as *ADAM11*, *RBFOX3*, and *HTR1E* may also indicate a favorable prognosis.

The same gene set also delineates a subgroup within LGG comprising both astrocytoma and oligodendroglioma patients. This subgroup appears to have a transitional profile between astrocytoma and oligodendroglioma, as 10 genes were found in a list of the top 100 genes upregulated in tumor with grade II compared to grade III, in *IDH1* mutated gliomas [34]—*ABLIM2*, *RYR2*, *ADAM11*, *RBFOX3*, *TMEM130*, *HTR1E*, *MPPED1*, *DMTN*, *MAP7D2* and *PACSIN1*. The other genes selected with them—*SLC12A5*, *DOC2A*, *NAPB*, *CPNE9*, *FADS6*, *MFSD4A*, *CAMKK1*, *SMIM10L2B*, *GFOD1*, *NEURL1*, *PHF24*, *CAMK2A*, *SNAP25*, *HIPK4*, *SULT4A1*, *PHYHIP*, *NCDN*, *DGKE*, *STX1A* and *CYP4X1*—are also upregulated in this group.

Astrocytoma and oligodendroglioma patients were well characterized. Astrocytomas showed high expression of immune-related genes, with 30 genes upregulated compared to oligodendrogliomas, 23 of which were significantly associated with survival (*SLC2A5*, *ADRB2*, *P2RY13*, *CSF2RA*, *SELPLG*, *IKZF1*, *ITGAM*, *LPAR5*, *SYK*, *RHBDF2*, *DOCK2*, *TMEM119*, *ATP8B4* and *TMEM52B*, *BHLHE41*, *LPCAT2*, *RGS10*, *RASAL3*, *ADAM28*, *IRF8*, *DEF6*, *WDFY4*, *PLCB2*, *APBB1IP* and *BLNK*). Interestingly, the less aggressive GBM subgroup shared this immune-like molecular profile with astrocytoma.

High methylation in oligodendroglioma affects genes linked to cell differentiation and immune function (for all regions and within promoter regions). Since similar genes are overexpressed in astrocytoma, this suggests methylation may silence them, indicating a possible interaction between these regulatory mechanisms. As noted in [29], promoter methylation is well known to be a key factor in gene silencing. In fact, several probes, eight of thirty, were located near the gene *ISM1*, which is known for its roles in anti-angiogenesis, immune response regulation, and the promotion of apoptosis—functions that highlight its anti-cancer potential [35]. However, methylation at these sites did not appear to directly regulate *ISM1*. Instead, a stronger correlation was observed with these immune-system function genes, including *SLC2A5*, *BLNK*, *TMEM119*, and *PLXDC2*. However, these alterations may not be driven solely by these epigenetic modifications, as mutations could also play a contributory role.

Overall, the three glioma types were well distinguished, although astrocytoma can be challenging to classify due to overlapping genetic profiles with both GBM and oligodendrogliomas.

## 5. Conclusions

In this study, we integrated genomics, epigenomics, and transcriptomics data from TCGA to investigate glioma heterogeneity using multi-omics factor analysis. The analyses were based on the most recent WHO 2021 classification, which served as the reference standard for distinguishing among astrocytoma, oligodendroglioma, and glioblastoma patients.

The identified factors combined information across the different omics layers, including genomics (mutations), epigenomics (DNA methylation), and transcriptomics (mRNA and miRNA), thereby reclassifying gliomas into five clusters, confirming numerous features previously associated with known groups while distinguishing between some glioma subtypes. For example, a distinct molecular profile within GBM, associated with the neural system and characterized by elevated expression of related genes, was identified, which may inform the development of novel prognostic markers and therapeutic strategies.

Supplementary analyses, including differential gene expression, gene set enrichment analysis, survival analysis, and Kaplan–Meier curves comparing the obtained groups, further corroborated the identified patterns at all the omics layers studied. Additionally, several novel features were identified that showed significant associations with survival.

The biomarkers identified in this study may help guide more personalized treatment strategies. Furthermore, given the distinct molecular profiles observed across glioma types, these biomarkers could be particularly valuable for improving disease classification, guiding treatment strategies, and enhancing prognostic assessments.

While these multi-omics profiles provide a robust framework for understanding glioma heterogeneity, our findings are primarily intended to serve as hypothesis-generating results. Future research utilizing independent, external validation is essential to validate these candidate biomarkers and confirm their clinical relevance and applicability. Future work could also re-annotate CpG probes and transcript identifiers using updated annotation resources and, where appropriate, integrate curated miRNA target databases to enable explicit miRNA–mRNA regulatory analyses. Additionally, the reduced sample overlap is a known limitation of retrospective multi-omics datasets; in our study, the final integrated cohort represents approximately 28% of the combined TCGA cases. Future studies using cohorts with more complete multi-assay coverage would improve statistical power and the generalizability of the findings. 

## Figures and Tables

**Table 1:** Clinical characteristics of patients in the “TCGA-GBM” and “TCGA-LGG” cohorts. Clinical characteristics of patients in the “TCGA-GBM” and “TCGA-LGG” cohorts. Values are also shown for the full dataset (may not sum to total due to missing data). Primary diagnosis reflects TCGA clinical annotation (legacy); WHO 2021 harmonized labels were used for all analyses.

		TCGA-GBM(*n* = 617)	TCGA-LGG(*n* = 516)	Total(*n* = 1133)
**Age, mean (SD)**		58 (14.41)	43 (13.36)	51 (15.78)
**Sex,** ***n*** **(%)**				
	Female	230 (37.3)	230 (44.6)	460 (40.6)
	Male	366 (59.3)	285 (55.2)	651 (57.5)
**Vital Status,** ***n*** **(%)**				
	Alive	101 (16.4)	388 (75.2)	489 (43.2)
	Dead	492 (79.8)	126 (24.2)	618 (54.5)
**Primary Diagnosis,** ***n*** **(%)**				
	Astrocytoma	-	194 (37.6)	194 (17.1)
	Oligodendroglioma	-	190 (36.8)	190 (16.8)
	Mixed Glioma	-	131 (25.4)	131 (11.6)
	Glioblastoma	599 (97)	-	599 (52.3)

**Table 2:** Concordance between TCGA and WHO glioma classifications. The table shows the number of samples classified under each category by TCGA (available in clinical data) compared with the corresponding current WHO classification (available in [22]).

		WHO			
		Astrocytoma	Glioblastoma	Oligodendroglioma	Unclassified
**TCGA**	Astrocytoma	130	41	5	18
	Glioblastoma	33	441	2	119
	Mixed Glioma	78	13	36	4
	Oligodendroglioma	41	11	128	10

**Table 3:** Pairwise log-rank test *p*-values between clusters for the 5-cluster solution.

	Cluster 1	Cluster 2	Cluster 3	Cluster 4
Cluster 2	$8.98\times 10^{-33}$	–	–	–
Cluster 3	$5.41\times 10^{-1}$	$3.85\times 10^{-25}$	–	–
Cluster 4	$1.32\times 10^{-11}$	$5.41\times 10^{-1}$	$1.05\times 10^{-9}$	–
Cluster 5	$2.66\times 10^{-13}$	$5.72\times 10^{-1}$	$7.12\times 10^{-10}$	$8.50\times 10^{-1}$

**Table 4:** Summary statistics for each identified cluster, including clinical and molecular characteristics. For each cluster, we report the number of patients, mean age, proportion of males, and proportion of deceased individuals. The last columns display the distribution of glioma subtypes and the prevalence (%) of some key mutations.

	Number of Patients	Age (Mean)	Male (%)	Dead (%)	ASTRO	GBM	OLIGO	IDH1 (%)	PTEN (%)	EGFR (%)	TP53 (%)
GBM-1	45	62.87	55.56	71.11	0	44	0	0	33.33	31.11	17.78
ASTRO-2	124	38.41	63.71	18.55	114	0	9	100	0	0	90.32
GBM-3	39	55.89	42.11	56.41	0	34	0	0	30.77	23.08	17.95
MIX-LGG-4	55	40.75	54.55	12.73	23	1	26	76.36	0	3.64	34.55
OLIGO-5	55	48.11	49.09	23.64	5	1	49	89.09	0	0	10.91

## Data Availability

The R code developed for this analysis is open source and available at https://github.com/sysbiomed/MOFA-in-Gliomas (accessed on 15 April 2026). The original datasets are not included due to their large size, but detailed instructions for downloading them are provided in the repository. The datasets are publicly available from The Cancer Genome Atlas (TCGA) database at https://portal.gdc.cancer.gov. The glioma classification using the WHO-2021 taxonomy guidelines is available at https://github.com/sysbiomed/MONET.
